# Efficacy and safety of colistin monotherapy versus colistin-based combination therapy in pediatric patients with multidrug-resistant gram-negative infections: A propensity score analysis

**DOI:** 10.1016/j.nmni.2026.101857

**Published:** 2026-09-22

**Authors:** Ajaree Rayanakorn, Peninnah Oberdorfer, Wasan Katip

**Affiliations:** a Department of Pharmacology, Faculty of Medicine, Chiang Mai University, Chiang Mai, 50200, Thailand; b Epidemiological and Innovative Research Group of Infectious Diseases (EIRGID), Chiang Mai University, Chiang Mai, 50200, Thailand; c Division of Infectious Diseases, Department of Pediatrics, Faculty of Medicine, Chiang Mai University, Chiang Mai, 50200, Thailand; d Department of Pharmaceutical Care, Faculty of Pharmacy, Chiang Mai University, Chiang Mai, 50200, Thailand

**Keywords:** Colistin, Multi-drug resistant, Gram-negative infection, Propensity score

## Abstract

**Objectives:**

To compare the efficacy and safety of colistin monotherapy versus colistin-based combination therapy in pediatric patients with multidrug-resistant Gram-negative bacteria (MDR-GNB) infections.

**Methods:**

This retrospective cohort analysis encompassed patients under the age of 18 years diagnosed with MDR-GNB infections who were administered intravenous colistin at Chiang Mai University Hospital during the period from 2010 to 2024. Inverse probability weighting (IPW) utilizing propensity scores was employed to equilibrate baseline characteristics. The primary endpoint was defined as 30-day mortality. Secondary endpoints comprised end-of-treatment mortality, clinical response, microbiologic response, and nephrotoxicity. Both univariable and multivariable logistic regression analyses were conducted subsequent to the implementation of propensity score weighting via IPW.

**Results:**

Colistin alone and combination therapy did not substantially affect 30-day mortality among 218 patients (74 monotherapy and 144 combination therapy) (adjusted odds ratio [aOR], 2.50; 95% confidence interval [CI], 0.69–9.15; p = 0.165). Clinical response (aOR, 1.27; 95% CI, 0.52–3.10; p = 0.595), end-of-treatment mortality (aOR, 0.79; 95% CI, 0.32–1.91; p = 0.595), and nephrotoxicity rate (aOR, 0.93; 95% CI, 0.40–2.17; p = 0.867) did not show any significant differences. When compared to monotherapy, patients receiving colistin-based combination therapy had a significantly greater microbiologic response rate (aOR, 3.62; 95% CI, 1.08–12.10; p = 0.037).

**Conclusions:**

Colistin-based combination therapy improved microbiologic response but showed no mortality or safety advantage over monotherapy in pediatric patients with MDR-GNB. Colistin monotherapy may provide comparable clinical outcomes without increased toxicity.

## Highlights

• Colistin-based combination therapy improved microbiological response in paediatric MDR-GNB infections.
• Mortality, clinical response, and nephrotoxicity were comparable between treatment groups.
• Propensity score–based IPW balanced baseline covariates and improved treatment effect estimation.
• Further studies are needed to determine optimal colistin treatment strategies in children.

## Graphical abstract

**Figure:**
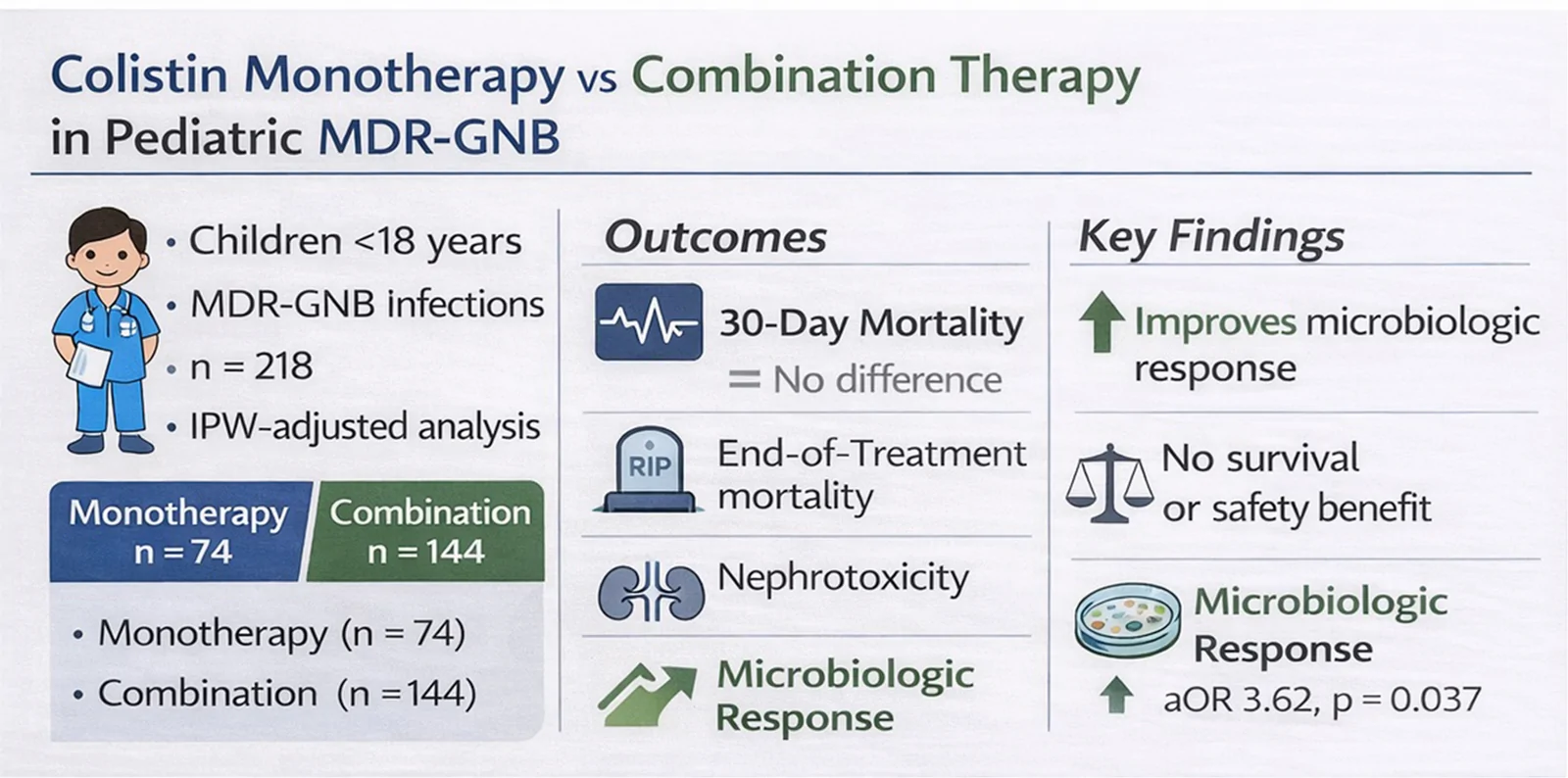

## 1 Introduction

The increasing prevalence of multidrug-resistant Gram-negative bacteria (MDR-GNB), including *Acinetobacter baumanni, Pseudomonas aeruginosa, Klebsiella pneumonia*, and *Carbapenem-resistant Enterobacteriaceae (CRE)* have posed major challenges in the management of hospital-acquired infections. These pathogens exhibit resistance to multiple antibiotic classes, rendering infections difficult to treat and contributing to high mortality rates ranging from 26 to 80% [1]. A meta-analysis reported that patients with MDR-GNB infections face a 1.78-fold higher risk of mortality compared to those with other infections [2].

The threat of antibiotic resistance is particularly pronounced in the WHO South-East Asia region [3,4]. In Thailand, approximately 19,000 additional deaths annually are attributable to multi-drug-resistant bacteria, with more than 80% of these associated with Gram-negative organisms [5]. In light of the limited therapeutic options, the WHO has designated the development of new treatments for MDR-GNB infections as a global research and development priority [6].

Over the past decade, carbapenems, especially meropenem have served as the mainstay of therapy either as alone or in combination with amikacin for treating infections caused by Gram-negative bacteria [7,8]. However, the widespread carbapenems use has led to the emergence of carbapenem-resistant strains. Consequently, older antibiotics such as colistin have been reintroduced to combat this growing threat. Colistin, also known as polymyxin E is a polypeptide antibiotic used for treatment of MDR-GNB infections with reported clinical cure rates of up to 89% in pediatric MDR-GNB infections [9]. Owing to its possession of both hydrophilic and lipophilic properties, it can act on the lipopolysaccharide component of Gram-negative bacterial membranes, leading to cell membrane disruption and bactericidal effects [10].

Nevertheless, the clinical efficacy of colistin monotherapy remains controversial. Due to its potent *in vitro* synergistic activity with carbapenems [[11], [12], [13]], combination therapy with colistin and carbapenems has been proposed to enhance treatment outcomes and mitigate resistance development. In addition to carbapenems, colistin has demonstrated *in vitro* and clinical synergistic potential when combined with other antimicrobial classes, including β-lactams such as cephalosporins and piperacillin/tazobactam, β-lactam/β-lactamase inhibitor combinations (e.g., sulbactam or ampicillin–sulbactam), fluoroquinolones, and fosfomycin [[11], [12], [13]].

These combinations are frequently employed in clinical practice in an attempt to maximize bacterial killing, reduce the emergence of resistance, and improve microbiological and clinical outcomes, particularly in infections caused by multidrug-resistant Gram-negative pathogens. Despite these recommendations, clinical studies comparing colistin monotherapy to colistin-based combination therapy have yielded inconsistent findings. Moreover, most available evidence is derived from adult populations, leaving a substantial knowledge gap regarding pediatric patients.

This retrospective study intends to close this gap by assessing the clinical effectiveness and safety of colistin monotherapy in comparison to colistin combination treatment in pediatric patients with MDR-GNB infections.

## 2 Methods

### 2.1 Study design and setting

The medical records of pediatric patients with multidrug-resistant Gram-negative bacteria (MDR-GNB) who were admitted to Chiang Mai University Hospital (CMUH), a 1400-bed tertiary teaching hospital, between 2010 and 2024 are the subject of this a retrospective study. The Research Ethics Committee at Chiang Mai University’s Faculty of Medicine approved the study (IRB no.015/2025). Because the study was retrospective and did not involve direct patient participation, informed consent was waived. All patient identifiers and personal information were removed and handled with strict confidentiality throughout the study procedures.

### 2.2 Study population and data collection

Pediatric patients aged 0–18 years who received intravenous colistin for ≥ 72 h with a confirmed diagnosis of multidrug-resistant Gram-negative bacterial (MDR-GNB) infection were included. MDR-GNB was defined as a Gram-negative organism resistant to at least three antibiotic classes with intrinsic activity against Gram-negative bacteria, including β-lactams, aminoglycosides, carbapenems, and quinolones [[14], [15], [16]].

Electronic medical records of pediatric patients admitted to Chiang Mai University Hospital (CMUH) with documented colistin use were reviewed (n = 519). Eligible cases were those with MDR-GNB isolated from blood, cerebrospinal fluid, sputum, or other clinically relevant specimens, accompanied by systemic signs of infection persisting for > 8 h, who had received intravenous colistin either as monotherapy or in combination with other antimicrobial agents for at least 72 h.

Patients were excluded if they were older than 18 years, had infections caused by other pathogens or Gram-negative bacterial colonization without clinical evidence of active infection, or received antimicrobial regimens that did not include colistin. Those who received colistin-based therapy for < 72 h or underwent renal replacement therapy (RRT) during the treatment course were also excluded (Fig. 1).

**Fig. 1:**
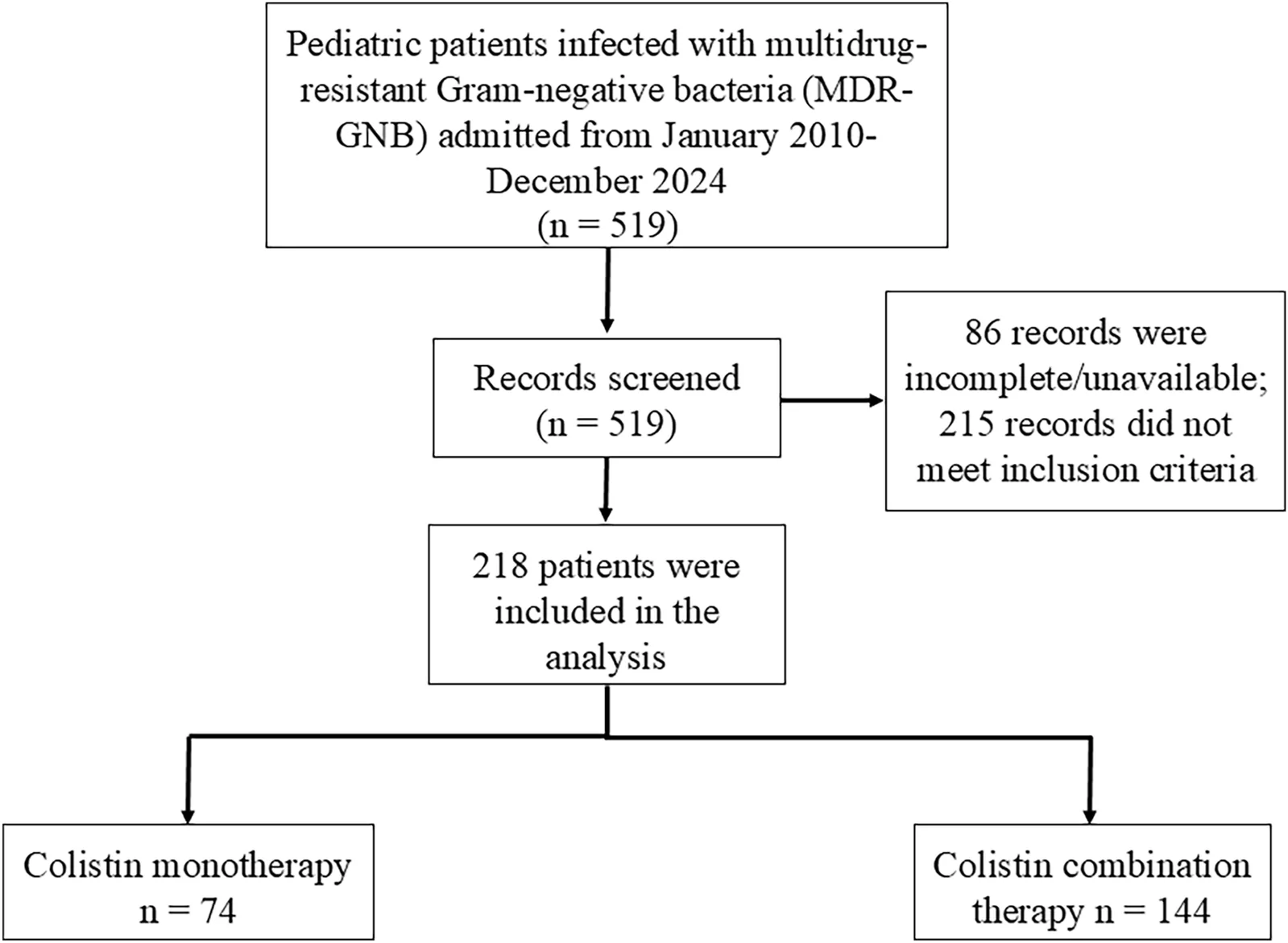
Flow diagram of the study population.

Patient demographics, medical history, ICU admission status, use of medical ventilation, comorbidities, Pediatric Glasgow Coma Scale (pGCS) score, Pediatric Sequential Organ Failure Assessment (pSOFA) score, Pediatric Index of Mortality 3 (PIM3), site of infection, concurrent nephrotoxic medications, length of hospital stay, clinical manifestations, laboratory results, antimicrobial treatment, susceptibility, and other relevant baseline characteristics were collected and analysed.

Colistin monotherapy was defined as intravenous colistin administered without concomitant systemic antibacterial agents with Gram-negative activity overlapping for ≥72 h during the colistin treatment course. Colistin-based combination therapy was defined as intravenous colistin administered concurrently with at least one additional systemic Gram-negative agent, with an overlap duration of ≥72 h (initiated within 24 h of colistin initiation). Eligible combination agents included carbapenems, cephalosporins, piperacillin/tazobactam, sulbactam-containing regimens, aminoglycosides, fluoroquinolones, and fosfomycin. Concomitant anti–Gram-positive agents (e.g., vancomycin) were recorded but were not considered combination agents for treatment-group classification. Colistin is administered as its inactive prodrug form, colistimethate sodium (also known as colistin methanesulfonate, CMS). The currently recommended CMS dosage is 2.5-5 mg of colistin base activity (CBS)/kg/day [17]. Based on recent pediatric pharmacokinetic data, a dose of 7.5 mg CBA/kg/day is suggested for children with serum creatinine (SCr) levels of 0.1–0.3 mg/dL, and 5 mg CBA/kg/day for those with SCr >0.3 mg/dL [18].

Concomitant nephrotoxic medications were recorded as potential confounders for nephrotoxicity assessment during the colistin treatment course, regardless of indication. These medications were not used to classify treatment groups, and some may have been prescribed for unrelated infections or other clinical conditions.

### 2.3 Study outcomes and outcomes definitions

The primary outcome of this study was the 30-day mortality rate, operationally defined as mortality from any etiology occurring within a 30-day period subsequent to the identification of the pathogen. The secondary outcomes encompassed mortality at the conclusion of the therapeutic intervention, clinical response, microbiological response, and nephrotoxicity. Mortality at the conclusion of treatment was operationally defined as any death occurring upon the finalization of the therapeutic regimen. Clinical response was assessed based on the resolution or partial resolution of fever, leucocytosis defined as an increased levels of leukocytes above the normal ranges [19], and local signs and symptoms related to MDR-GNB infections. Microbiological response was defined as the absence of MDR-GNB in at least one follow-up culture obtained from the originally infected site after an initial positive culture. In cases where follow-up cultures were not available, microbiological response was inferred from surrogate clinical indicators, including hospital discharge, clinical improvement, or discontinuation of antibiotic therapy. Nephrotoxicity is defined in accordance with the 2009 consensus guidelines regarding vancomycin, which specify that it is identified by an increase in serum creatinine (SCr) levels of ≥ 0.5 mg/dL or a rise of ≥ 50% from baseline, as observed on two consecutive measurement occasions [20], or, alternatively, by the more sensitive Risk, Injury, Failure, Loss, and End-stage Kidney (RIFLE) criteria [21].

### 2.4 Statistical analysis

The baseline characteristics of the patients were summarized using descriptive statistics based on the distribution’s normality. For normally distributed data, continuous variables were expressed as mean ± standard deviation (SD); for non-normally distributed data, values were written as median (interquartile range, IQR). Frequencies and percentages were used to display categorical variables. For categorical variables, the Chi-square (χ2) test was used to compare groups. For continuous variables, the Student’s t-test was applied to normally distributed data, whereas the Mann–Whitney *U* test was used for nonparametric comparisons. A p-value < 0.05 was considered statistically significant.

Patients were categorized into two treatment groups according to the regimen received: colistin monotherapy or colistin-based combination therapy. For patients with multiple infection episodes, only the first occurrence of MDR-GNB infection was included in the analysis.

To address baseline imbalances and reduce potential confounding, propensity score weighting using the inverse probability of treatment weighting (IPTW) method was applied. Propensity scores were estimated via multivariable logistic regression incorporating variables potentially influencing outcomes including sex, age, pGCS, pSOFA, PIM3, presence of septic shock, ICU status, vasoactive drug use, baseline serum creatinine, ventilator-associated pneumonia, and bacteraemia. Potentially collinearity was also checked for covariate selection. Each patient was then weighted by the inverse of their propensity score to achieve covariate balance between treatment groups.

Univariate logistic regression was used to analyze primary (30-day mortality) and secondary outcomes (clinical response, mortality at the end of treatment, and microbiological response), followed by multivariable logistic regression to calculate adjusted odds ratios (aORs) and 95% confidence intervals (CIs). Inverse probability weighting (IPTW) using the propensity score was employed to adjust baseline covariates. Covariate balance before and after IPTW adjustment was assessed using the standardized mean difference (SMD), with an absolute SMD > 10% indicating meaningful imbalance [22]. All statistical tests were two-tailed, with significance defined as p < 0.05.

### 2.5 Sample size estimation

Sample size estimation was based on the in-hospital mortality rates reported by Abdelsalam et al. which were 43.3% for the colistin monotherapy group and 16.7% for the colistin–meropenem combination group [23]. Assuming a two-sided α = 0.05 and 80% power (β = 0.20), the minimum required sample size was calculated to be 92 patients.

### 2.6 Antimicrobial susceptibility testing

All clinical isolates were identified and tested for antimicrobial susceptibility in the hospital microbiology laboratory using standard laboratory procedures. Bacterial identification was performed using routine conventional biochemical methods and an automated identification system according to the laboratory’s standard operating procedures.

Antimicrobial susceptibility testing was conducted using an automated susceptibility testing system. Colistin susceptibility was determined using the Sensititre system (Broth-Based). Antimicrobial susceptibility results were interpreted according to CLSI guidelines. For colistin, CLSI interpretive criteria classify isolates as Intermediate (I) or Resistant (R) (no susceptible category is provided). Colistin MIC ranges by treatment group are presented in Table 1. The susceptibility of *A. baumannii* isolates exhibiting multiple antibiotic resistance and multidrug-resistant Gram-negative bacteria (MDR-GNB) was evaluated. Identification and antimicrobial susceptibility testing were performed using the automated system VITEK 2 (bioMérieux, Marcy-l’Étoile, France). The results were interpreted according to the guidelines of the Clinical and Laboratory Standards Institute (CLSI) [24]. Multidrug-resistant (MDR) Gram-negative bacteria were defined as isolates non-susceptible to at least one agent in three or more antimicrobial classes. The antimicrobial susceptibility profiles were used to guide the selection of colistin monotherapy or colistin-based combination therapy at the discretion of treating physicians.

**Table 1:** Baseline characteristics of patients receiving colistin monotherapy versus colistin-based combination therapy before inverse probability of treatment weighting.

Characteristics	Overall cohort n = 218		
	Colistin monotherapy (n = 74)	Colistin combination therapy (n = 144)	P value
Age (years±SD)	7.46 ± 6.68	4.72 ± 5.36	0.001
Infants (>1 month–1 year), n (%)	19 (25.68)	52 (36.11)	0.120
Children (>1–12 years), n (%)	29 (39.19)	67 (46.53)	0.301
Adolescents (>12 years), n (%)	26 (35.14)	25 (17.36)	0.003
Male, n (%)	49 (46.80)	89 (91.20)	0.522
Pediatric Glasgow Coma Scale (pGCS), median (IQR)	11.50 (11-15)	11 (11-15)	0.867
pSOFA	4.76 ± 0.45	5.63 ± 0.33	0.123
Pediatric Index of Mortality 3 (PIM3), median (IQR)	2.15 (1.30-4.60)	3.10 (1.90-7.35)	0.043
Mechanical ventilation, n (%)	63 (85.14)	127 (88.19)	0.523
Septic shock, n (%)	12 (26.09)	28 (33.33)	0.035
ICU status, n (%)	61 (82.43)	129 (89.58)	0.135
Length of stay, median (IQR)	58.50 (32-105)	47 (32-88.5)	0.266
Site of infection, n (%)			
Lung	51 (68.92)	103 (71.53)	0.689
Blood	6 (8.11)	26 (18.06)	0.049
Urine	5 (6.76)	4 (2.78)	0.162
Cerebrospinal fluid	1 (1.35)	3 (2.08)	0.703
Intra-abdominal	2 (2.70)	3 (2.08)	0.772
Skin and soft tissue	8 (10.81)	4 (2.78)	0.014
Surgical site infection	1 (1.35)	1 (0.69)	0.630
Principle diagnosis, n (%)			
Hospital-acquired pneumonia (HAP)	34 (45.95)	45 (31.25)	0.033
Ventilator-associated pneumonia (VAP)	15 (20.27)	57 (39.58)	0.004
Sepsis	8 (10.81)	26 (18.06)	0.163
Infected wound	7 (9.46)	5 (3.47)	0.066
Others^a^	10 (13.51)	11 (7.64)	0.164
Comorbidities n (%)			
Chronic Kidney Disease	6 (8.50)	19 (16.50)	0.264
Congenital heart disease	17 (22.10)	48 (42.90)	0.113
Congenital hypotonia	0	1 (0.7)	0.472
Congenital pneumonia	4 (2.40)	3 (4.60)	0.188
Hypertension	1 (2.70)	7 (5.30)	0.192
Patent ductus arteriosus (PDA)	11 (9.20)	16 (17.80)	0.426
Respiratory distress syndrome (RDS)	7 (10.50)	24 (20.5)	0.149
Cancer	15 (11.5)	19 (22.50)	0.173
Systemic lupus erythematosus (SLE)	1 (1.70)	4 (3.30)	0.505
Gastrointestinal disorders	11 (9.80)	18 (19.20)	0.626
Down’s syndrome	3 (3.10)	6 (5.90)	0.968
Others¶	10 (12.2)	26 (23.8)	0.392
Type of nephrotoxic medications^b^			
Amikacin	31 (41.89)	51 (35.42)	0.350
Amphotericin B	26 (35.14)	42 (29.17)	0.368
Digoxin	14 (18.92)	28 (19.44)	0.926
Gentamicin	3 (4.05)	4 (2.78)	0.613
Vancomycin	51 (68.92)	109 (75.69)	0.284
Rifampicin	1 (1.35)	0	0.162
Vasopressor use	25 (33.78)	72 (50.00)	0.023
Baseline Serum creatinine, mg/dL, median (IQR)	0.60 (0.32-0.87)	0.40 (0.28-0.60)	0.024
Receiving colistin loading dose	1 (1.35)	9 (6.25)	0.102

Note:

a Others: meningitis, Urinary tract infection (UTI), Intra-abdominal infection, Infected pancreatic pseudocyst, Community-acquired pneumonia (CAP); Others¶: pulmonary candidiasis, malnutrition, coagulopathy, Omenn syndrome, systemic juvenile idiopathic arthritis, decompensated cirrhosis, hemophagocytic lymphohistiocytosis, congenital stenosis of trachea, digeorge syndrome, kwashiorkor, congenita laryngomalacia.

b Nephrotoxic medications represent concomitant medications administered during the colistin treatment course and were not necessarily part of the MDR-GNB treatment regimen or used for treatment-group classification.

## 3 Results

A total of 218 patients were included in the analysis, comprising 74 patients who received colistin monotherapy and 144 patients who received colistin-based combination therapy. The mean age of patients in the colistin monotherapy group was 7.46 ± 6.68 years and, compared with 4.72 ± 5.36 years in the combination therapy group (p = 0.001). Males accounted for nearly half of the colistin monotherapy group (n = 49, 46.8%) and most of the combination therapy group (n = 89, 91.2%). The baseline Pediatric Index of Mortality 3 (PIM3) score was slightly lower in the monotherapy group compared with the combination therapy group (2.15 [1.30–4.60] vs. 3.10 [1.90–7.35], p = 0.043). Septic shock was observed in approximately one-fourth of patients receiving monotherapy and one-third of those receiving combination therapy (n = 12, 26.1% vs. n = 28, 33.3%, p = 0.035). The lungs were the most common sites of infection, followed by bloodstream and skin/soft tissue. A higher proportion of bloodstream infections was observed among patients receiving colistin-based combination therapy (n = 26, 18.1% vs. n = 6, 8.1%, p = 0.049). Ventilator-associated pneumonia (VAP) was more frequent in the combination therapy group than in the monotherapy group (n = 57, 39.6% vs. n = 15, 20.3%, p = 0.004). Similarly, the use of vasoactive drugs was higher among patients receiving combination therapy compared with those receiving monotherapy (n = 72, 50.0% vs. n = 25, 33.8%, p = 0.023). The baseline serum creatinine of colistin based combination therapy was slightly lower in the combination therapy group than in the monotherapy group (0.40 [0.28–0.60] vs. 0.60 [0.32–0.87], p = 0.024). No significant differences were observed in comorbidities or the use of nephrotoxic medications between groups (Table 1). *Acinetobacter baumannii* was the most common causative pathogen, and the most frequently used combination regimen was colistin with a carbapenem, particularly meropenem (Table 2).

**Table 2:** Causative microorganisms and treatment regimen.

Characteristics	Overall cohort n = 218	
	Colistin monotherapy (n = 74)	Colistin combination therapy (n = 144)
Main pathogen, n (%)		
*Acinetobacter Baumannii*	61 (82.45)	121 (83.49)
*Pseudomonas aeruginosa*	9 (12.16)	11 (9.17)
*Klebsiella pneumonia*	3 (4.05)	8 (5.05)
Others‡	1 (1.35)	4 (2.29)
Treatment regimen, n (%)		
Colistin	74 (100.00)	
Colistin + meropenem		95 (62.80)
Colistin + cephalosporin‡		15 (9.90)
Colistin + piperacillin/tazobactam		14 (9.20)
Colistin + sulbactam		7 (4.86)
Colistin + ampicillin-sulbactam		4 (2.60)
Colistin + levofloxacin		2 (1.30)
Colistin + fosfomycin		1 (0.70)
Others§		6 (4.17)
Colistin MIC rang (mg/L)	0.01-1	0.01-1

*Note:* ‡Others: Enterobacterales (CRE), *Chryseobacterium indologenes, Enterobacter Cloacae*, *Elizabethkingia anophelis*, *Alcaligenes* spp; §Others: Amikacin, imipenem ‡Cephalosporin: ceftazidime, cefoperazone in combination with sulbactam.

The two treatment groups’ baseline characteristics were balanced by applying propensity score weighting using IPW. The details of potential baseline characteristics that could affect treatment outcomes were listed in Fig. 2. Before applying inverse probability weighting (IPW), substantial imbalances were observed between treatment groups across several prognostic factors, as reflected by the larger standardized differences (|SMD| > 0.1) in variables such as age, pSOFA, PIM3, septic shock, ICU status, ventilator-associated pneumonia (VAP), use of vasoactive drugs, and bacteremia.

**Fig. 2:**
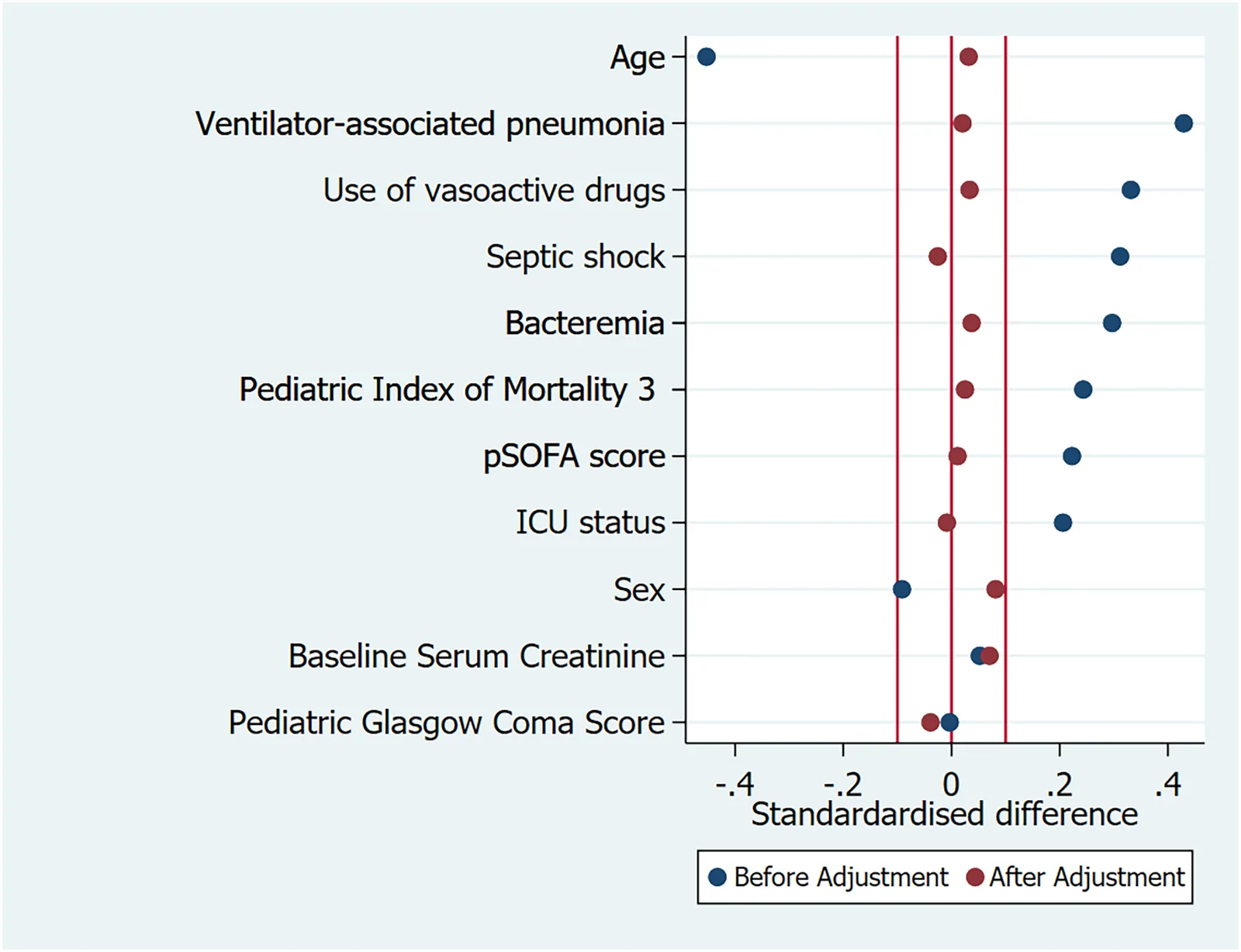
Standardized difference of baseline characteristics by treatment groups before and after inverse probability treatment weighting.

After IPW adjustment, the standardized differences of all variables were notably reduced, with all weighted standardized differences falling below 0.1, indicating adequate covariate balance between treatment groups. The standardized differences for most variables approached zero, suggesting that the weighting procedure effectively minimized baseline imbalances (Fig. 2).

Before weighting, colistin based combination therapy was not significantly associated with 30-day mortality, end-of-treatment mortality, clinical response, microbiological response or nephrotoxicity rate with a non-significant trend toward higher microbiological response, but this was not statistically significant (Table 3). After adjusting for confounders using IPW, colistin combination therapy was not associated with significant differences in 30-day mortality (adjusted odds ratio [aOR], 2.50; 95% confidence interval [CI], 0.69–9.15; p = 0.165), mortality at the end of treatment (aOR, 0.79; 95% CI, 0.32–1.91; p = 0.595), clinical response (aOR, 1.27; 95% CI, 0.52–3.10; p = 0.595), or nephrotoxicity rate (aOR, 0.93; 95% CI, 0.40–2.17; p = 0.867) compared with monotherapy. Nevertheless, compared to monotherapy, combination therapy was substantially associated to increased likelihood of microbiological response (aOR, 3.62; 95% CI, 1.08–12.10; p = 0.037) (Table 4).

**Table 3:** Association between colistin combination therapy and outcomes in critically ill pediatric patients before inverse probability of treatment weighting.

Outcomes and variable	Logistic regression analysis					
	Monotherapy n = 74 (%)	Combination n = 144 (%)	OR (95%CI)	P-value	aOR (95% CI)	p-value
Primary outcome						
30-day mortality rate	3 (4.05)	12 (8.33)	2.15 (0.59-7.88)	0.247	2.94 (0.63-13.89)	0.172
Secondary outcomes						
End of treatment mortality	11 (14.86)	35 (24.31)	1.84 (0.87-3.87)	0.109	1.14 (0.46-2.84)	0.770
Clinical response	61 (82.43)	105 (72.92)	0.54 (0.26-1.15)	0.109	0.87 (0.35-2.16)	0.770
Microbiological response	65 (87.84)	133 (92.36)	1.67 (0.66-4.24)	0.277	2.69 (0.91-7.98)	0.075
Nephrotoxicity	16 (21.62)	38 (26.39)	1.30 (0.67-2.53)	0.441	0.83 (0.36-1.94)	0.673

Confidence interval; aOR, adjusted odds ratio; pGCS, pediatric Glasgow Coma Scale; pSOFA, pediatric Sequential Organ Failure Assessment; PIM3, Pediatric Index of Mortality 3; ICU, intensive care unit. The adjusted model included sex, age, pGCS, pSOFA, PIM3, septic shock, ICU status, vasoactive drug use, baseline serum creatinine, ventilator-associated pneumonia, and bacteremia.

**Table 4:** Association between colistin combination therapy and outcomes in critically ill pediatric patients after inverse probability of treatment weighting.

Outcomes and variable	Propensity score (IPW)			
	Logistic regression analysis			
	OR (95%CI)	P-value	aOR (95% CI)	p-value
Primary outcome				
30-day mortality rate	1.75 (0.43-7.06)	0.435	2.50 (0.69-9.15)	0.165
Secondary outcomes				
End of treatment mortality	0.85 (0.37-1.94)	0.697	0.79 (0.32-1.91)	0.595
Clinical response	1.18 (0.51-2.70)	0.697	1.27 (0.52-3.10)	0.595
Microbiological response	**3.13 (1.11-8.81)**	**0.031**	**3.62 (1.08-12.10)**	**0.037**
Nephrotoxicity	1.00 (0.49-2.07)	0.981	0.93 (0.40-2.17)	0.867

IPW, Inverse probability weighting; CI, confidence interval; aOR, adjusted odds ratio; pGCS, pediatric Glasgow Coma Scale; pSOFA, pediatric Sequential Organ Failure Assessment; PIM3, Pediatric Index of Mortality 3; ICU, intensive care unit. The adjusted model included sex, age, pGCS, pSOFA, PIM3, septic shock, ICU status, vasoactive drug use, baseline serum creatinine, ventilator-associated pneumonia, and bacteremia.

## 4 Discussion

In order to treat multidrug-resistant Gram-negative bacterial (MDR-GNB) infections, this retrospective cohort study examined the efficacy of colistin monotherapy against colistin-based combination therapy. The findings indicate that while combination therapy does not appear to reduce mortality or improve clinical outcomes compared with monotherapy, it may offer an advantage in achieving microbiological clearance without increasing toxicity. The fact that statistical significance emerged only following IPTW adjustment suggests that balancing baseline severity and clinical characteristics is essential to uncover this potential treatment effects.

The absence of substantial discrepancies pertaining to mortality rates between the two treatment cohorts aligns with findings from earlier investigations [25,26]. This underscores the scarcity of conclusive evidence regarding the most effective treatment modalities and the complexities involved in the management of CRE infections. A systematic review assessing the efficacy of antibiotic interventions for CRE infections in a cohort of 692 patients revealed no statistically significant differences in mortality outcomes between the combination therapy and monotherapy groups, with the exception of three studies encompassing 194 critically ill patients with bacteremia, which indicated potential advantages of combination therapy in select populations. Nevertheless, the authors identified methodological shortcomings and considerable heterogeneity among the studies included, which necessitated the execution of further rigorously designed randomized trials [27].

After adjustment for confounders, most clinical effectiveness outcomes did not differ significantly between treatment strategies. However, colistin-based combination therapy was associated with a significantly higher likelihood of microbiological response compared with monotherapy (aOR 3.62; 95% CI 1.08–12.10; p = 0.037). Although this did not translate into improved survival or overall clinical response, it suggests enhanced bacterial eradication, potentially reflecting synergistic activity between colistin and carbapenems, particularly against *Acinetobacter baumannii*, the predominant causative organism in this cohort [28].

This finding aligns with a systematic review and meta-analysis by Wang et al. [29], which found no significant differences in clinical response or mortality beyond 28 days between monotherapy and combination therapy, but demonstrated improved microbiological eradication with combination regimens (OR 0.49; 95% CI 0.32–0.74; *p* = 0.0009) [29].

The discrepancy between the observed improvement in microbiological response and the absence of corresponding clinical or survival benefits may be explained by several factors. Outcomes in critically ill pediatric patients are often influenced by multiple determinants, including host immune response, organ dysfunction, baseline disease severity, and the overall burden of comorbidities, which may attenuate the clinical impact of enhanced bacterial clearance. In addition, limited penetration of colistin into pulmonary tissue and the possibility that microbiological clearance occurred too late to alter the trajectory of critical illness may further reduce the likelihood of survival benefit. Residual confounding is inherent to observational analyses may also have contributed to these findings. These observations are consistent with prior studies in adult populations, in which improved microbiological outcomes or *in vitro* synergy have not consistently translated into meaningful survival advantages [30].

Nephrotoxicity remains a major concern with colistin use, particularly in pediatric patients. In this study, no significant difference in nephrotoxicity was observed between colistin monotherapy and combination therapy after IPW adjustment (aOR 0.93; 95% CI 0.40–2.17; p = 0.867). Importantly, baseline serum creatinine was slightly higher in the monotherapy group prior to adjustment, and the use of other nephrotoxic medications was comparable between groups. The absence of increased nephrotoxicity in the combination therapy group suggests that adding a second agent, particularly carbapenems such as meropenem, does not appear to exacerbate renal toxicity beyond that associated with colistin itself. This finding supports existing evidence that colistin-associated nephrotoxicity is primarily dose-dependent and related to patient-specific risk factors rather than synergistic toxicity from combination regimens. From a clinical perspective, this is reassuring, as clinicians may consider combination therapy for microbiological benefit without substantially increasing the risk of renal injury.

Taken together, these findings suggest that in pediatric with MDR-GNB infections, colistin-based combination therapy may improve microbiological eradication but does not confer additional survival or clinical response benefits compared with monotherapy. Importantly, this enhanced microbiological response is achieved without increasing nephrotoxicity risk. Therefore, the decision to use combination therapy should be individualized, potentially reserved for cases where rapid bacterial clearance is critical (e.g., bloodstream infections or VAP due to *A. baumannii*), while recognizing that such an approach may not necessarily improve mortality outcomes.

Notably, the present study included a heterogeneous population of MDR-GNB infections, encompassing a range of pathogens and clinical conditions. This heterogeneity reflects real-world practice but may limit the comparability of outcomes across subgroups. In addition, our study included all MDR-GNB infections based on internationally accepted definitions, rather than being restricted to carbapenem-resistant organisms. As combination therapy is most commonly recommended for carbapenem-resistant pathogens, caution is warranted when extrapolating these findings to that subgroup. Therefore, the results should be interpreted with caution, and further studies focusing on more homogeneous populations, particularly carbapenem-resistant infections, are needed.

Numerous limitations must be taken into account when evaluating the results of this study. First, this was a retrospective observational study conducted in a single tertiary-care center, which may limit the external validity and generalizability of our findings. Furthermore, although the cohort represents one of the largest pediatric studies in this field, the sample size remained relatively modest, potentially limiting the statistical power to detect differences in mortality and safety outcomes. Although propensity score weighting using IPW was applied to minimize baseline imbalances, residual confounding from unmeasured variables cannot be completely excluded. Factors such as timing of antibiotic initiation, appropriateness of dosing, source control, and clinician decision-making processes were not fully captured and may have influenced treatment outcomes. Although time-to-event analyses may provide additional information regarding survival dynamics, complete and reliable event-time data were not available for all patients because of the retrospective study design. Therefore, logistic regression was selected to evaluate fixed-time outcomes. In addition, although antimicrobial susceptibility testing (AST) was routinely performed to guide clinical care, isolate-level susceptibility results were not consistently available in our retrospective database. Therefore, we were unable to systematically assess whether individual patients received microbiologically appropriate (*in vitro* active) therapy in either treatment group, which may have introduced residual confounding. Second, there were substantial differences in baseline severity between groups prior to weighting, with patients receiving combination therapy presenting with more severe clinical conditions (e.g., higher PIM3 scores, more septic shock, VAP, vasoactive drug use, and bacteremia). Although IPW successfully balanced measured covariates, this statistical adjustment cannot fully replicate the effect of randomization, and treatment selection bias may persist. Third, the relatively small sample size, particularly in the colistin monotherapy group, limited the statistical power to detect differences in important outcomes such as mortality and nephrotoxicity. This is reflected in the wide confidence intervals observed in several adjusted odds ratios (e.g., 30-day mortality aOR 2.50; 95% CI 0.69–9.15), suggesting imprecision in effect estimates. Fourth, heterogeneity in the combination regimens may have influenced the outcomes. Although carbapenems, particularly meropenem, were the most common agents used in combination, a range of other antibiotics was also administered. Some combinations may be of limited clinical relevance in this context. As the study did not stratify outcomes according to specific combination partners, the reported microbiological benefit may not be generalizable across all colistin-based combination regimens. Finally, the findings may have limited generalizability to other settings, particularly institutions with different antimicrobial resistance patterns, prescribing practices, and patient populations. The predominance of *Acinetobacter baumannii* in this cohort may also limit applicability to infections caused by other multidrug-resistant Gram-negative pathogens.

## 5 Conclusion

Colistin-based combination therapy was associated with better microbiological outcomes in pediatric patients with MDR-GNB infections but showed similar 30-day mortality, mortality at the end of treatment, nephrotoxicity, and clinical response rates compared to colistin monotherapy. However, given the retrospective observational design, limited sample size, and clinical heterogeneity of the cohort, these findings should be interpreted cautiously and considered hypothesis-generating. According to these results, colistin monotherapy may yield comparable clinical outcomes to combination therapy in this cohort, despite the observed difference in microbiological response. Therefore, the decision to initiate combination therapy should be individualized, taking into account infection severity, the causative pathogen, and the broader clinical context. To determine the best treatment approaches for pediatric populations, larger multicenter prospective studies, ideally randomized controlled trials, are needed to confirm these findings and better define optimal treatment strategies.

## CRediT authorship contribution statement

**Ajaree Rayanakorn:** Writing – review & editing, Writing – original draft, Software, Resources, Project administration, Methodology, Investigation, Funding acquisition, Formal analysis, Data curation, Conceptualization, Validation, Visualization. **Peninnah Oberdorfer:** Validation, Supervision, Data curation. **Wasan Katip:** Writing – review & editing, Writing – original draft, Validation, Supervision, Methodology, Formal analysis, Conceptualization.

## Ethical approval and informed consent

The study was approved by the Research Ethics Committee, Faculty of Medicine, Chiang Mai University (IRB no.015/2025). Informed consent was waived due to a retrospective nature of the study without direct patient involvement. All patient identifiers and personal information were removed and handled with strict confidentiality throughout the study procedures.

## Funding

This work was supported by the 10.13039/501100010731Faculty of Medicine, Chiang Mai University (Grant No. 103-68), and 10.13039/501100002842Chiang Mai University.

## Declaration of competing interest

The authors declare that they have no known competing financial interests or personal relationships that could have appeared to influence the work reported in this paper.
